# Effect of retinoic acid on the tight junctions of the retinal pigment epithelium-choroid complex of guinea pigs with lens-induced myopia *in vivo*

**DOI:** 10.3892/ijmm.2014.1651

**Published:** 2014-02-10

**Authors:** SHA WANG, SHUANGZHEN LIU, JUNFENG MAO, DAN WEN

**Affiliations:** Department of Ophthalmology, Xiangya Hospital, Central South University, Changsha, Hunan 410008, P.R. China

**Keywords:** retinoic acid, Zonula occludens-1, occludin, lens-induced myopia, guinea pigs

## Abstract

Zonula occludens-1 (ZO-1) and occludin are important tight junction (TJ)-associated proteins, which are expressed in the retinal pigment epithelium (RPE)-choroid complex. Retinoic acid (RA) is a regulator of eye growth and may play an important role in forming functional TJs. The aim of this study was to detect the changes that occur in the expression of ZO-1 and occludin in the RPE-choroid complex of guinea pigs with lens-induced myopia (LIM), and to investigate the effect of RA on TJ-associated proteins *in vivo*. We developed an animal model of myopia by placing a −6.00 D negative lens on the right eyes of 3-week-old guinea pigs. The refractive error and axial length of the eye were measured on days 0, 3, 7 and 14. High-performance liquid chromatography (HPLC) was performed to detect the changes in endogenous RA in the RPE-choroid complex. The expression of ZO-1 and occludin was observed by immunofluorescence and assayed by western blot analysis. Additionally, 2 μl LE540 (2.5 μg/μl), an antagonist of RA receptors (RARs), was injected into the vitreous chamber of the eyes of guinea pigs with LIM and 2 μl phosphate-buffered saline (PBS) (2.5 μg/μl) were injected as a negative control. We observed no obvious change in RA, ZO-1 and occludin expression in the normal control group within 14 days. In the LIM and LIM plus PBS groups, the level of RA and the expression of ZO-1 and occludin in the RPE-choroid complex significantly increased within 14 days along with the development of myopia. However, the level of RA was inhibited and the expression of TJ-associated proteins decreased in the eyes of guinea pigs with LIM following the injection of LE540. Thus, we consider that the expression of ZO-1 and occludin is increased in the RPE-choroid complex during the development of myopia. This change in expression may be regulated by RA, a factor known to be involved in the regulation of eye growth.

## Introduction

Human myopia primarily results from the abnormal elongation of the vitreous chamber of the eye (1). A similar eye elongation associated with myopia can readily be induced in eyes of monkeys (2), tree shrews (3), marmosets (4) and guinea pigs (5), by depriving them of form vision or wearing negative lenses. A great deal of research has focused on the starting point of the development of myopia, the retina, and the end point, the sclera (6–9). There is little research on the retinal pigment epithelium (RPE) that may be the connection between the retina and the sclera. The RPE separates the outer layer of the neural retina from the capillaries of the choroid to form the outer blood-retinal barrier. It is the first cell type to differentiate in the retina, but as the neural retina and choroid develop around it, 40% of the RPE transcriptome will change its expression (10). The RPE is also crucial for maintaining the microenvironment of the sensory retina and the choriocapillaris (11).

Retinoic acid (RA) is a biologically active regulator that has a broad range of functions, including cell differentiation, proliferation and apoptosis in various cell types (12,13). It has been suggested to be a chemical signal involved in the regulation of ocular growth (14–18). Studies in various species have reported that both the concentration of RA and the expression of RA receptor-β (RAR-β) exhibit bidirectional and reversible changes in the retina and choroid during the development of myopia.

Zonula occludens-1 (ZO-1) is a 210–225 kDa protein found at the submembranous domain of tight junctions (TJs) in the epithelium and endothelium. At the TJ, ZO-1 is associated with the carboxyl terminal end of claudins through its first PDZ domain (19), and through its second and third PDZ domain to JAM (20), and by its GK module to occludin (21). Numerous studies employing cytokines, hormones and growth factors have found the abundance of ZO-1 to be associated with the degree of tightness of the junction. Occludin, another TJ-associated protein, was originally discovered in avian tissues by Furuse *et al* (22), using anti-chick occludin antisera prepared in rats. It was found to be localized to epithelial and endothelial TJs, and was subsequently confirmed as the first integral membrane TJ protein to be identified.

Recent studies have shown that RA may promote the function of the epithelial barrier, and its bioavailability regulates the epithelial barrier, which is accompanied by altering the expression of TJ-associated proteins (23). The aim of this study was to investigate the changes in the expression of TJ-associated proteins in the RPE-choroid complex in the eyes of guinea pigs with lens-induced myopia (LIM), and to investigate the effect of RA on the TJs of the RPE-choroid complex of guinea pigs *in vivo*.

## Materials and methods

### Establishment of animal model of myopia and animal housing

**Figure 1:**
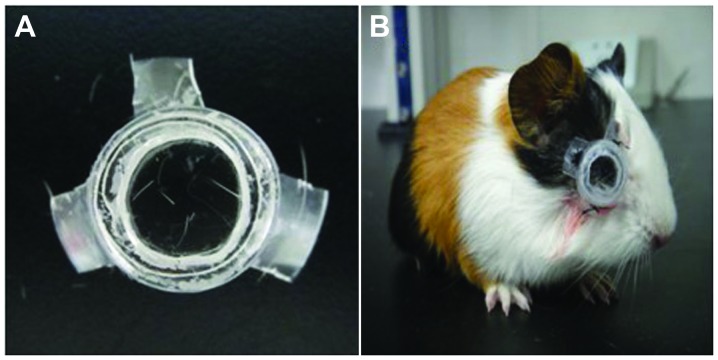
The guinea pig model of lens-induced myopia. (A) A single use Murphy dropper was trimmed to create a frame for the lens. (B) The self-made spectacle was sutured and fixed to the right eye of the guinea pig.

Sixty clean pigmented guinea pigs 3 weeks of age were obtained from the Animal Department of the Central South University, Changsha, China. A concave optical resin lens of −6.00 D was provided by Hong Kong Optical Lens Co., Ltd., Hong Kong, China. The lens diameter was 12 mm and the inner arc curvature was 9.61 mm. A single use Murphy dropper was trimmed to make a frame for the lens (Fig. 1A). Two small apertures were left at the bottom of the frame for cleaning and air circulation. Pentobarbital sodium (0.3%) at a dosage of 30 mg/kg was injected into the abdominal cavity of the guinea pigs for anesthesia, and the hairs around the fissura orbitalis were sheared. The self-made frame was sutured and fixed to the soft tissue around the fissura orbitalis of the right eye (Fig. 1B). Guinea pigs were randomly divided into 4 groups: group A, normal control group (n=15, no lens on each eye); group B, LIM group (n=15, −6.00 D optical lens on the right eye); group C, LIM plus phosphate-buffered saline (PBS)-injected group (n=15, PBS was injected into the vitreous chamber of the eye with LIM); and group D, LIM plus LE540-injected group (n=15, LE540 was injected into the vitreous chamber of the eye with LIM). The animals were housed in plastic boxes (60×40×20 cm) with wire mesh lids. The boxes contained a small hiding shelf at one end (32×16×14 cm) and were lined with wood shavings. Water (supplemented with vitamin C) and food (guinea pig pellets, hay and occasionally, fresh vegetables) were freely available. Lighting was provided by ceiling fluorescent tubes (36 W) on a 12-h light/dark cycle (lights on at 10 am). The room temperature was kept at 22°C. The treatment and care of the animals were conducted according to the ARVO Statement for the Use of Animals in Ophthalmic and Vision Research, and were approved by the Committee for Animal Welfare of the Xiangya Hospital of Central South University.

### Biometric measurements

Tropicamide (0.25%) was administered 3 times at 5-min intervals to dilate the pupil. Refractive error (RE) measurements were obtained using streak retinoscopy 45 min after the first mydriasis. Axial length was measured with a CineScan A/B ultrasonographic machine (Optikon 2000 S.p.A., Rome, Italy; sensitivity, 0.01 mm). A topical anesthesia (0.5% proparacaine hydrochloride; Alcon, Puurs, Belgium) was administered prior to the ultrasound measurement. The ultrasound probe was placed in direct contact with the cornea during the axial measurement. RE were presented as the mean spherical equivalent refractive error (MSE). Due to the powerful accommodation ability of guinea pig eyes, a retinoscopy was conducted in the central zone shortly after the ciliary muscle was completely paralyzed. The measurements above were performed on days 0, 3, 7 and 14 after lense placement. All refraction data were presented as the means derived from 5 repeated measurements.

### Collection of RPE-choroid complex samples

The guinea pigs were sacrificed on days 0, 3, 7 and 14 when necessary with an overdose of pentobarbital sodium. The eyes were removed and dissected along the ora serrata, and were subsequently washed immediately in Hank’s balanced salt solution (HBSS; Gibco, Grand Island, NY, USA) with penicillin and streptomycin (200 μg/ml penicillin/streptomycin), and gentamicin sulfate (400 μg/ml) (all from Invitrogen, Carlsbad, CA, USA). After dissection of the anterior part of the eye, and the vitreous and neural retina, the RPE-choroid complex was carefully removed from the sclera. The procedures were performed on ice and under subdued lighting.

### Endogenous levels of RA after visual manipulations

The level of endogenous RA in the RPE-choroid complex was determined using high-performance liquid chromatography (HPLC). The RPE-choroid complex from 4 eyes was pooled and homogenized with a Polytron homogenizer (Brinkmann, Westbury, NY, USA) in 2 ml of water. The all-trans RA (atRA) standard was purchased from the Sigma-Aldrich (C_20_H_28_O_2_; molecular mass, 300.44). A Waters uBondapak C18 reversion phase chromatography column (150×3.9 mm) was used and the chromatographic conditions were as follows: mobile phase, V (acetonitrile):V (0.1% glacial acetic acid solution), 86:14; flow rate, 1.0 ml/min; detected wave length, 350 nm; column temperature, 25°C; sample size, 20 μl. The RA contents (ng) in the RPE-choroid complex/100 mg were calculated.

### Western blot analysis

Western blot analysis was perforemd to detect the protein expression of ZO-1 and occludin in the RPE-choroid complex. The RPE-choroid complex of the guinea pig eyes was collected and lysed in lysis buffer (150 mM NaCl, 50 mM Tris-HCl, pH 7.4, 2 M MEDTA, 1% NP-40) containing protease inhibitors (Boehringer, Mannheim, Germany). Total protein was resolved by SDS polyacrylamide gel electrophoresis, and then was transferred onto a nitrocellulose membrane. The membrane was incubated at 4°C overnight with rabbit anti-ZO-1 polyclonal antibody (1:250 dilution; no. 61–7300; Invitrogen) and mouse anti-occludin monoclonal antibody (1:500 dilution; no. 33–1500; Invitrogen). Peroxidase-conjugated secondary antibodies were used as secondary detection reagents with an enhanced chemiluminescence kit (GE Healthcare, New York, NY, USA). Chemiluminescent signals were visualized by exposure to X-ray film. Band intensities were quantified with BandScan software (version 5.0). Levels of GAPDH were used for standardization. The relative expression of the target protein was calculated. Independent experiments were performed, and repeated 3 times.

### Indirect immunofluorescence

Immunofluorescence was carried out to detect the distribution of ZO-1 and occludin proteins in the RPE-choroid complex in all groups. Briefly, fixed tissues were washed 3 times with PBS, covered with 10% normal donkey serum diluted in PBS, and incubated for 20 min at 37°C. Rabbit anti-ZO-1 polyclonal antibody was used at a 1:100 dilution (no. 40–2200; Invitrogen) and mouse anti-occludin monoclonal antibody at a 1:250 dilution (no. 33–1500; Invitrogen). PBS was used as a control for the primary antibody. Following overnight incubation with the primary antibody at 4°C temperature, the slides were rinsed 3 times with PBS and AlexaFluor 488 was added at a dilution of 1:500 for 1 h at 37°C.

### Statistical analysis

All data are presented as the means ± SEM. Statistical analyses used repeated measures (RM) or one-way ANOVA (SPSS 11.0) as specified. Post hoc analyses used Tukey’s least significant difference (LSD) test. A P-value <0.05 was considered to indicate a statistically significant difference.

## Results

### Effects of negative lens on the ocular refractive state

RE and axial lengths of all left eyes in each group developed at a normal rate. The right eyes developed differently in each group. In group A, refraction developed with respect to the direction of emmetropization. The REs and axial lengths of the right eyes did not differ significantly as compared to the left eyes at each time point (P>0.05). In group B and C, the diopter developed in the direction of myopia as time progressed in the eyes of guinea pigs with LIM. The most relative myopia was approximately 4.7 D on the 14th day, and differed significantly compared to the opposite eyes and normal control eyes (P<0.05) (Table I). Similarly, the axial length of the eyes of guinea pigs with LIM extended more rapidly than that in the contralateral eyes. However, the development of myopia and the speed of axial length growth in the eyes of guinea pigs with LIM were inhibited following the injection of LE540 into the vitreous, and there was statistically significant difference as compared with group B and C on the 7th and 14th day (P<0.05) (Table I).

### Effect of vision manipulation on the level of RA in the RPE-choroid complex

The level of RA exhibited no significant change in group A, and there was no difference observed between the right and left eyes (P>0.05). The level of RA increased with time in group B and C. In group B and C, the RA content in the RPE-choroid complex of the eyes of guinea pigs with LIM was 12.40±0.31 ng/100 mg and 12.33±0.23 ng/100 mg, respectively before LIM, and significantly increased to 137.85±1.02 ng/100 mg and 132.09±0.44 ng/100 mg on the 14th day. Moreover, the difference in the RA level was also significant between the right and left eyes on days 3, 7 and 14 (all P<0.05) (Table II). In group D, the RA level in the eyes of guinea pigs with LIM decreased from 12.18±01.23 ng/100 mg to 2.55±0.18 ng/100 mg on the 14th day after the injection of LE540, which was significantly lower than the level observed in the normal control eyes or contralateral eyes at the same time point. There was a statistically significant difference as compared to group B and C on days 3, 7 and 14 (all P<0.05) (Table II).

### Effect of vision manipulation on TJ-associated proteins in the RPE-choroid complex as determined by western blot analysis

**Figure 2:**
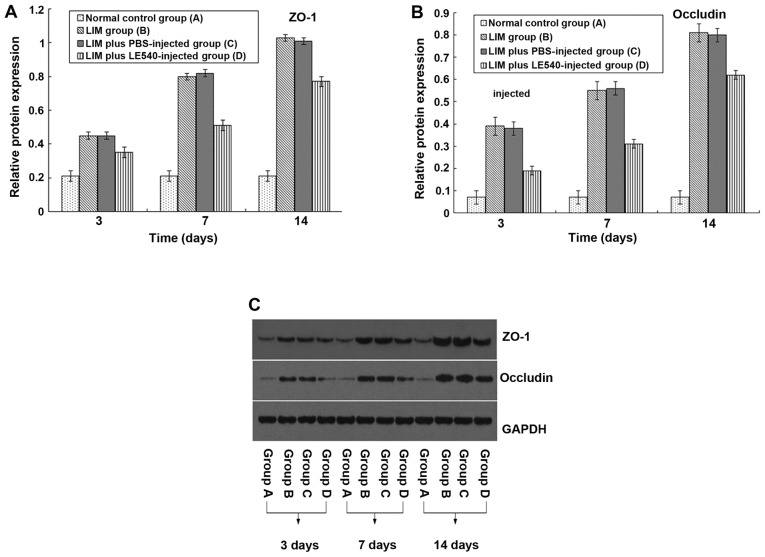
Change in tight junction-associated protein expression detected by western blot analysis. (A and B) Zonula occludens-1 (ZO-1) and occludin showed a similar trend in expression; no significant change in expression was observed in group A. An upregulated expression was observed in group B and C compared to group A (P<0.05). This upregulation was significantly inhibited in group D compared to groups B and C (P<0.05). (C) Protein electrophotogram of ZO-1 and occludin.

The expression of ZO-1 and occludin in the normal control group showed no obvious change (Fig. 2). After LIM, ZO-1 and occludin protein expression was upregulated in the RPE-choroid complex within 14 days and the differences on days 3, 7 and 14 were statistically significant between groups B and C and group A (P<0.05). By contrast, the increase in ZO-1 and occludin expression was partly inhibited by the injection of LE540, and there was a statistically significant difference when compared with the eyes of guinea pigs with LIM with or without the PBS injection (P<0.05).

### Effect of vision manipulation on TJ-associated proteins in the RPE-choroid complex as determined by immunofluorescence

**Figure 3:**
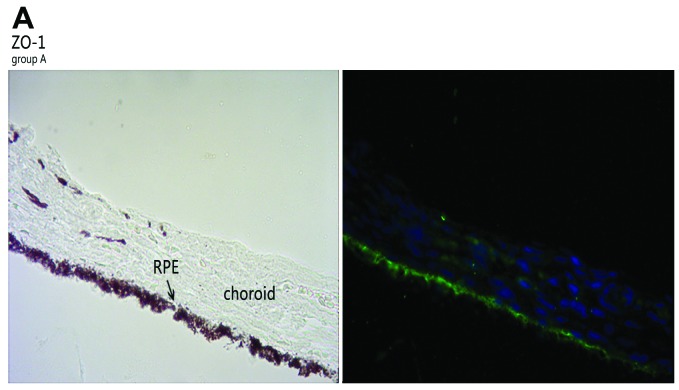

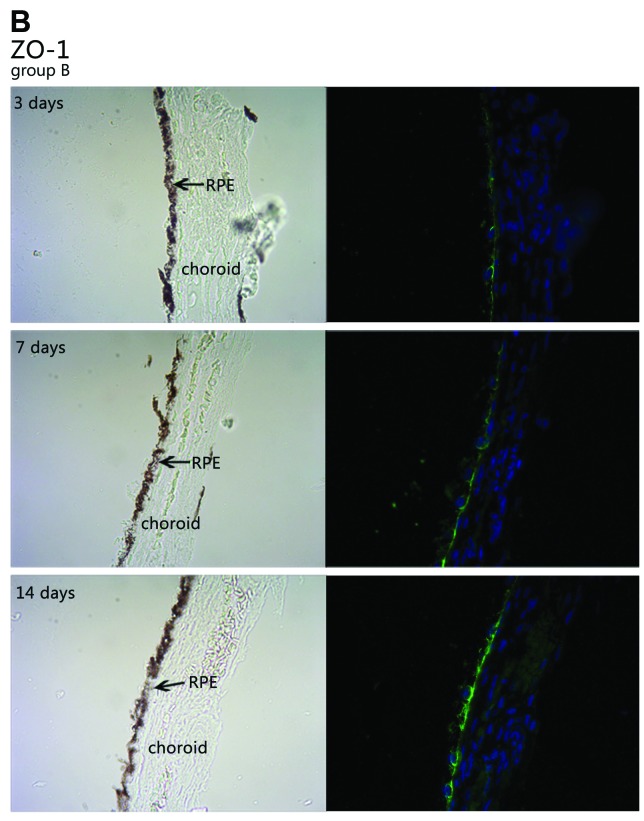

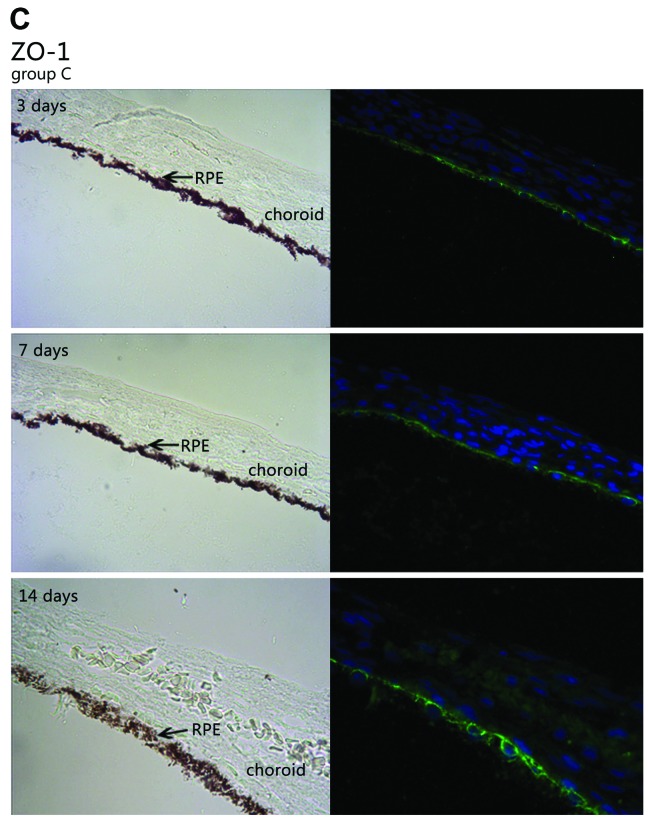

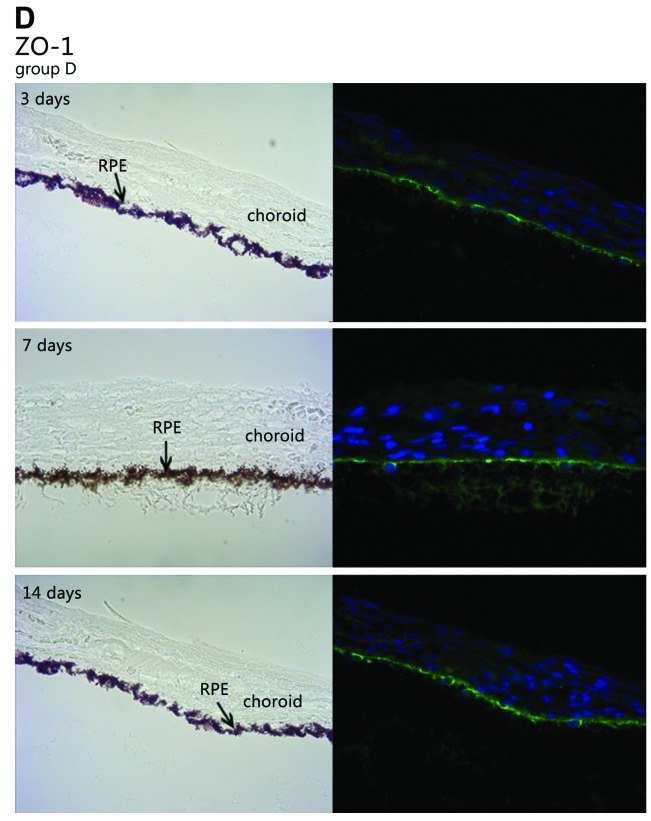

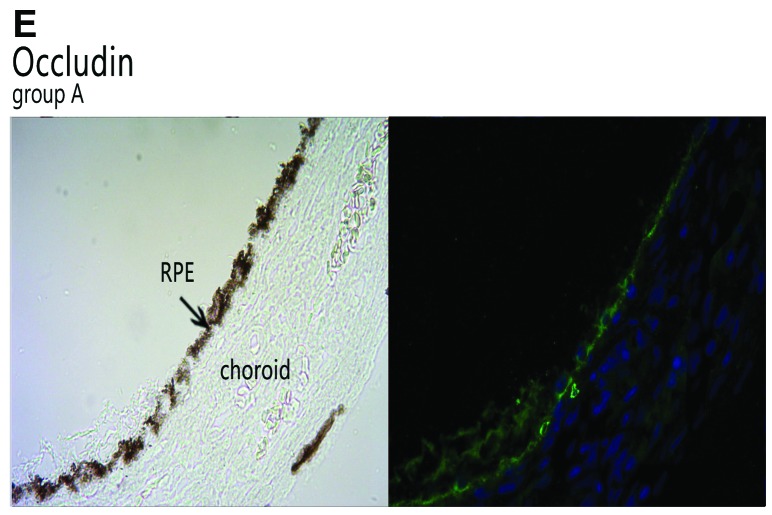

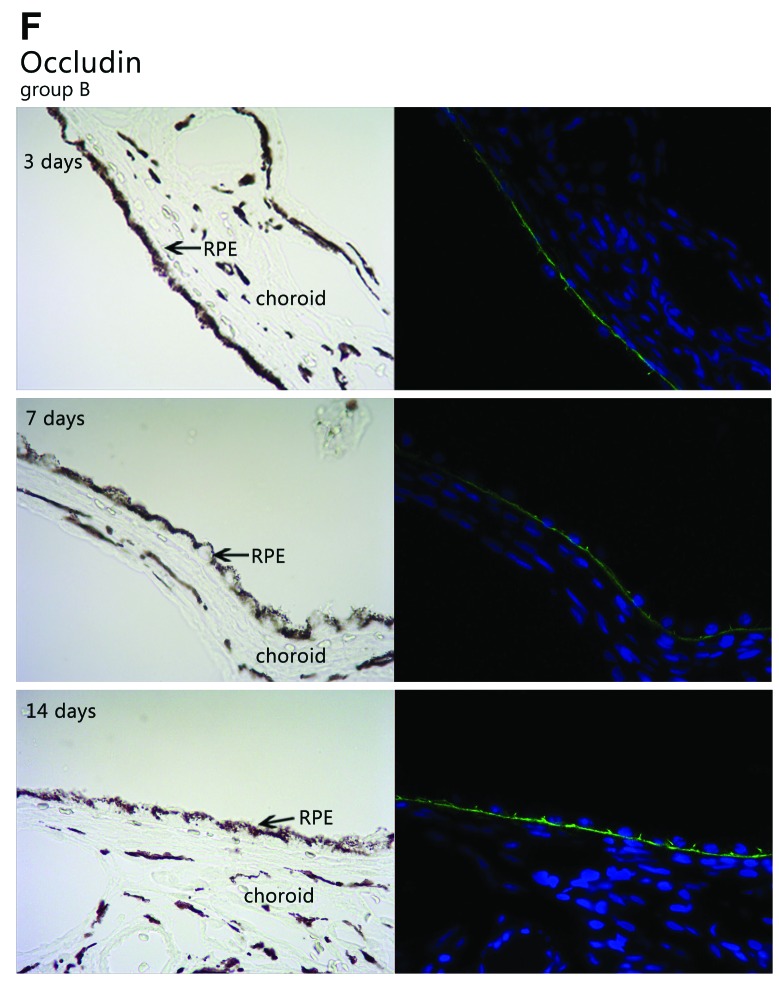

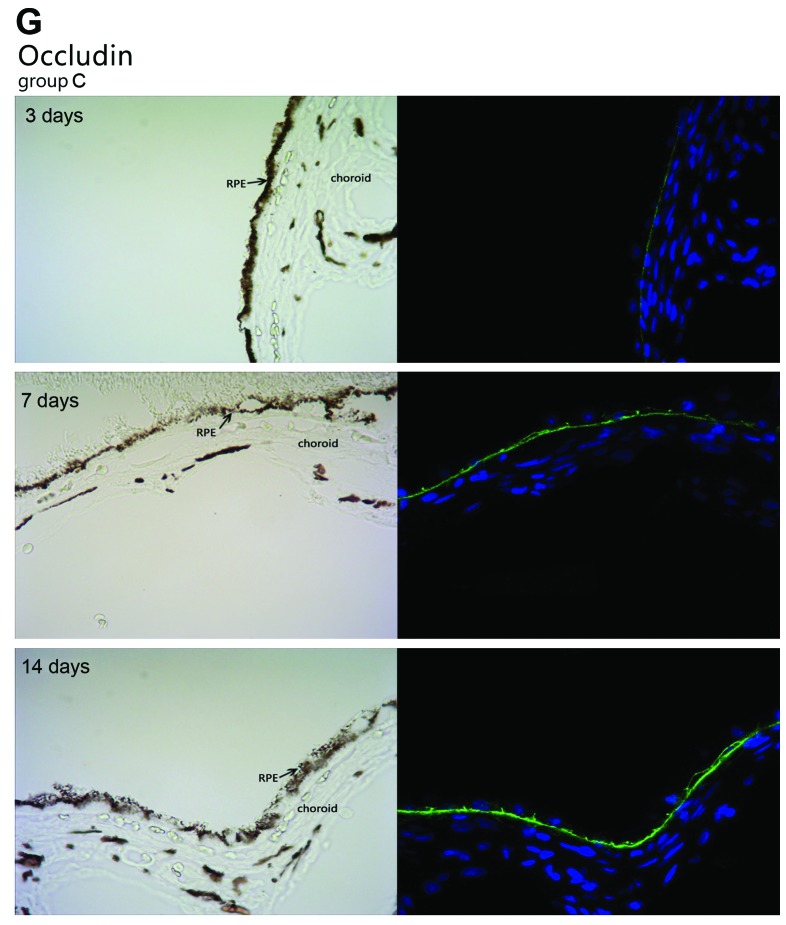

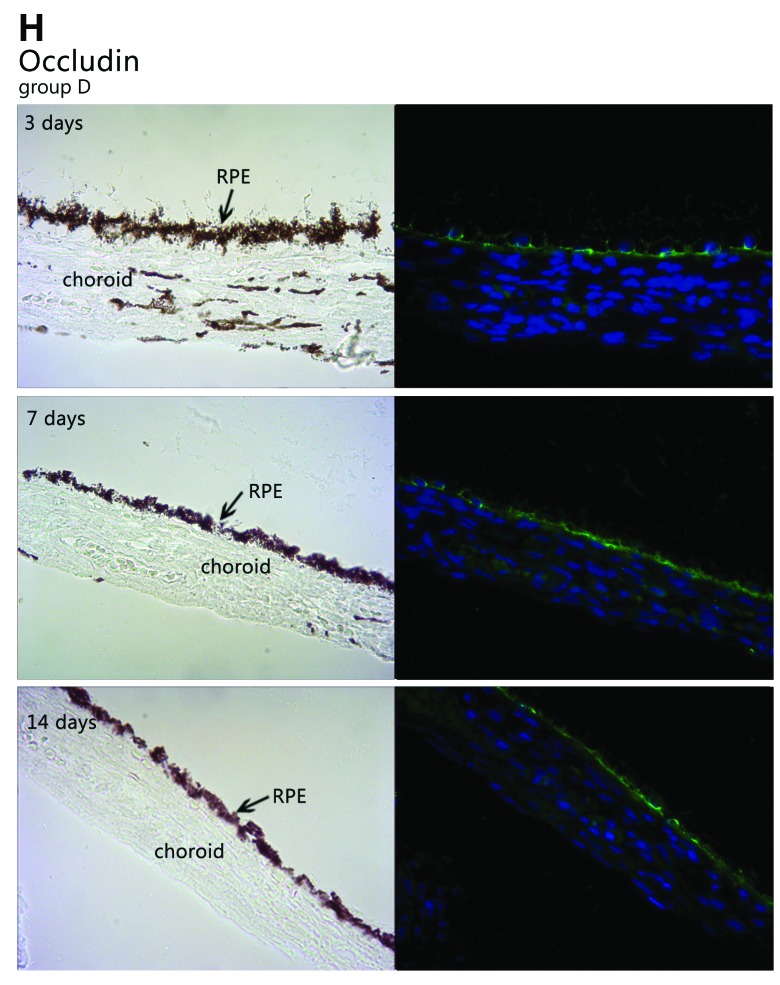
Immunofluorescence-stained images of serial cross sections of the retinal pigment epithelium (RPE)-choroid complex of guinea pigs. Compared with the normal control, an increase in Zonula occludens-1 (ZO-1) and occludin protein expression in the RPE was observed in the eyes of guinea pigs with lens-induced myopia (LIM) and this increase was suppressed in the LE540-injected group. (A and E) Expression and localization of ZO-1 and occludin in normal control group. (B and F) Expression and localization of ZO-1 and occludin at different experimental time points in group B. (C and G) Expression and localization of ZO-1 and occludin at different experimental time points in group C. (D and H) Expression and localization of ZO-1 and occludin at different experimental time points in group D.

In accordance with the results from western blot analysis, ZO-1 and occludin were expressed in the RPE (Fig. 3). The 2 proteins were upregulated in group B and C. The expression levels of TJ-associated proteins varied slightly at the different time points in group A. In the eyes of the guinea pigs with LIM, the immunostaining of ZO-1 and occludin was most intense in the RPE and choroid; this was not observed in the normal control group. After the injection of LE540, the expression of ZO-1 and occludin decreased.

## Discussion

A large amount of research has focused on the pathogenesis of myopia and RA has been determined to be a factor in the development of myopia. Seko *et al* (24) reported that RA levels were increased in the retina of chicks with form-deprived myopia. Merts and Wallman (16) reported that the synthesis of choroidal RA is modulated by those visual manipulations that influence ocular elongation and that this RA may reach the sclera in concentrations adequate to modulate scleral proteoglycan formation. However, the results of the association between RA and myopia have varied according to the species examined. Previously, McFadden *et al* (25) found that feeding RA to chickens can accelerate the speed of eye elongation and they concluded that RA may act at the level of a non-visual mechanism which regulates ocular growth. In this study, the level of RA in the RPE-choroid complex of the eyes of guinea pig was upregulated by wearing a negative lens. These results were consistent with those from the study by McFadden *et al* (25), namely that the level of RA was upregulated in the choroid during the development of myopia. On the contrary, the increase in the RA level was partly inhibited and the development of myopia was much slower when LE540, an antagonist of RARs (26), was injected into the vitreous chamber of the eyes of guinea pigs with LIM.

TJs that are synthesized and assembled during epithelial differentiation are the most apical structures of the junctional complex. They serve as a barrier to regulate the flow of solutes and fluid from the choroidal vasculature into the outer retina, and to control the pathway of ions and small molecules through paracellular channels. Occludin and claudins are linked to the cytoskeleton by the intracellular membrane-associated guanylate kinase homologs, ZO-1, ZO-2, ZO-3 and claudin-1 (27). The combination of claudin-1 and occludin is required for the establishment of an effective paracellular barrier (28). Numerous studies that have employed cytokines, hormones and growth factors have shown that the ZO-1 level is associated with the degree of tightness of the junction. The results from this study demonstrated that ZO-1 and occludin were upregulated in the RPE-choroid complex in the eyes of guinea pigs with LIM. Thus, we hypothesized that the TJs were reinforced by the 14th day in the eyes of guinea pigs with LIM. The reason for this finding is uncertain, but RA may be a regulator of TJ-associated proteins. Based on detection in F9 cells, in a colitis model, and in some cancer tissues (29–31), RA is believed to be an obligatory component in the differentiation of epithelial cells that leads to the establishment of epithelial integrity. In their study, Rong and Liu (23) observed that the expression of ZO-1 and occludin increased in ARPE-19 cultures treated with atRA, suggesting that atRA has a barrier function in a process involving a specific increase in these TJ-associated proteins. Of note, in this study, the increase in the expression of ZO-1 and occludin in the eyes of guinea pigs with LIM was partly inhibited following the injection of LE540 into the vitreous chamber. These results led us to hypothesize that although RA may play an important role in forming functional TJs, many other factors also regulate the expression of TJ-associated proteins during the development of myopia.

Myopia induced by negative lenses may be related to the myopia clinically observed in young humans who spend many hours reading, suggesting that insufficient accommodation (the ‘lag of accommodation’) also imposes hyperopic defocus. The majority of researchers have concluded that local modulation is the key factor in the development of myopia. This suggests that the neural retina itself has to be the source of growth-regulating signals, and that the sclera is the target of these signals. Thus, the RPE-choroid complex may play a critical role in signal transduction as a whole system. In this study, we found that both RA and TJ-associated proteins in the RPE-choroid complex were affected by optical manipulation in guinea pigs. However, it is not clear as to why the TJs were upregulated in the eyes of the guinea pigs with LIM and whether there is an association between RA and TJ-associated proteins. RA had been reported as a possible mediator of the changes in eye growth (15). It has been reported that retinal atRA levels are increased in myopic eyes with accelerated elongation, and decreased in eyes with inhibited elongation (32). As previously demonstrated, functional atRA can regulate the permeability of various cell types *in vitro*, and impaired atRA signaling leads to the disruption of functional TJs (29–31). It has been reported that changes in the blood-retinal barrier (BRB) are observed at 45 days after form deprivation, which suggests that abnormal BRB function is secondary to the development of myopia rather than the cause of myopia. The results of this study suggest that RA may play a protective role in the integrity of the TJs in the RPE-choroid complex, but whether its association with this complex is direct or indirect remains to be elucidated.

In the present study, when myopia developed, the vitreous chamber of the eyes of the guinea pigs became longer. Generally speaking, the barrier of the TJs may be disrupted due to the elongated axial length. However, we found that the TJs were enhanced rather than disrupted in this study, and the TJ-associated proteins in the RPE-choroid complex were upregulated. We speculate there are two possible reasons for this finding: i) TJs may have a compensatory mechanism within 14 days of the induction of myopia. The RPE cell number would not change, but the expansion of individual RPE cells and the increment of TJs between epithelial cells were most likely the responses to the ‘pulling force’ applied to RPE cells. ii) RA may play a protective role with respect to the TJs in the RPE-choroid complex. The upregulation of RA may be a negative feedback mechanism for the regulation of ocular growth, which led to the enhancement of TJs. However, if the vitreous chamber was excessively elongated, the TJs cannot compensate and RA cannot protect the integrity of the TJs.

The results of the present study indicate that RA is beneficial to TJs in the RPE-choroid complex. We speculate that the disruption of TJ function during the development of myopia is likely not the cause of myopia, but only the pathological phenomena of high myopia. These findings may be helpful for further research regarding the pathogenesis of myopia.

## Figures and Tables

**Table I:** Effect of negative lens on the ocular refractive state (n=15).

Groups	Time point (day)	Refraction (D)		Axial length (mm)	
	
		R	L	R	L
A: Normal control group (n=15)	0	3.14±0.71	3.21±0.55	7.58±0.07	7.56±0.06
	3	2.60±0.80	2.68±0.49	7.62±0.10	7.60±0.08
	7	2.01±0.65	2.07±0.43	7.65±0.04	7.64±0.05
	14	1.39±0.74	1.42±0.61	7.68±0.10	7.69±0.09
B: LIM group (n=15)	0	3.15±0.82	3.18±0.49	7.59±0.05	7.56±0.04
	3	1.93±0.80	2.68±0.49	7.64±0.11	7.61±0.04
	7	−0.21±0.65^a^,^b^	2.07±0.43	7.73±0.03^a^,^b^	7.64±0.07
	14	−3.29±0.74	1.42±0.61	7.88±0.09^a^,^b^	7.68±0.06
C: LIM plus PBS-injected group (n=15)	0	3.13±0.67	3.15±0.43	7.60±0.05	7.58±0.08
	3	1.90±0.80	2.65±0.39	7.64±0.05	7.62±0.06
	7	−0.23±0.56^a^,^b^	2.05±0.53	7.74±0.04^a^,^b^	7.64±0.05
	14	−3.27±0.44^a^,^b^	1.44±0.71	7.89±0.10^a^,^b^	7.67±0.06
D: LIM plus LE540-injected group (n=15)	0	3.18±0.45	3.21±0.57	7.58±0.11	7.57±0.05
	3	2.54±0.39	2.64±0.40	7.63±0.08	7.61±0.08
	7	1.82±0.37^a^,^c^	2.07±0.35	7.66±0.06^c^	7.63±0.07
	14	0.85±0.56^a^,^c^	1.46±0.49	7.71±0.08^c^	7.68±0.09

a P<0.05 vs. the left eye;

b P<0.05 vs. normal control group;

c P<0.05 vs. LIM group and LIM plus PBS-injected group.

LIM, lens-induced myopia; PBS, phosphate-buffered saline; R, right eye; L, left eye.

**Table II:** Levels of retinoic acid (RA) in the retinal pigment epithelium (RPE)-choroid complex after different vision manipulations (n=4).

		RA content (ng/100 mg)	
		
Groups	Time point (day)	R	L
A: Normal control group (n=15)	0	12.17±0.22	12.17±0.24
	3	12.20±0.20	12.19±0.19
	7	12.18±0.28	12.16±0.16
	14	12.21±0.18	12.20±0.27
B: LIM group (n=15)	0	12.40±0.31	12.37±0.29
	3	18.15±0.34^a^	12.34±0.35
	7	46.71±0.40^a^	13.01±0.35
	14	137.85±1.02^a^	12.67±0.40
C: LIM plus PBS-injected group (n=15)	0	12.33±0.23	12.39±0.30
	3	19.06±0.36^a^	12.41±0.39
	7	48.68±0.56^a^	12.47±0.34
	14	132.09±0.44^a^	12.50±0.29
D: LIM plus LE540-injected group (n=15)	0	12.18±0.23	12.21±0.27
	3	5.24±0.24^a^,^b^	12.19±0.30
	7	4.05±0.27^a^,^b^	12.17±0.25
	14	2.55±0.18^a^,^b^	12.23±0.29

a P<0.05 vs. the left eye and the normal control group;

b P<0.05 vs. LIM group and LIM plus PBS-injected group.

LIM, lens-induced myopia; PBS, phosphate-buffered saline; L, left eye; R, right eye.
